# Clinical effects of laparoscopic surgery for the treatment of endometriosis and endometriosis-fertility: A retrospective study of 226 cases

**DOI:** 10.3389/fsurg.2022.1049119

**Published:** 2023-01-16

**Authors:** Haiyan Li, Yingxue Han, Yuru Cai, Xiaojuan Su, Lixia Tan

**Affiliations:** Department of Gynecology, Shijiazhuang People’s Hospital, Shijiazhuang, China

**Keywords:** endometriosis, laparoscopic treatment, improvement of infertility, postoperative pregnancy, gynecology

## Abstract

**Introduction:**

To determine the clinical effects of laparoscopic surgery (LS) in the treatment of endometriosis and endometriosis-fertility.

**Methods:**

Two hundred twenty-six patients with endometriosis who underwent LS (LS group, *n* = 176) or laparotomy (LT group, *n* = 50) at the Third Hospital of Shijiazhuang City from June 2011 to June 2013 were included in this study, and their clinical outcomes for endometriosis and infertility were compared. All patients were followed up for 1 year after surgery to determine postoperative pregnancies in patients with endometriosis.

**Results:**

The operative times between the LS and LT groups were not significantly different (*P* > 0.05); however, the length of stay in the hospital and blood loss in the LS group were significantly different from the LT group (*P* < 0.05). The incidence of postoperative symptoms were lower in the LS group than the LT group (*P* < 0.05). The postoperative pregnancy rates in the two groups were significantly different, including the infertility patients (*P* < 0.05).

**Conclusions:**

Compared with LT, LS significantly reduced pain and improved the quality of life in women with endometriosis. These results can provide a reference for the clinical treatment of endometriosis.

## Introduction

Endometriosis (EM) is a common gynecologic condition characterized by the presence of endometrial tissue on the mucosa outside the uterine cavity. EM generally includes three distinct forms: superficial endometriosis, endometriomas (ovarian), and deep infiltrating endometriosis. EM may be located on the surface of the pelvic peritoneum, ovaries, ovarian cysts, or between the rectum and vagina (1). EM can lead to inflammatory reactions, resulting in clinical manifestations, such as lower abdominal pain, irregular menstruation, painful sexual intercourse, and dysmenorrhea (2). In addition, EM is the main cause leading to infertility in women (3). EM is responsible for 25%–30% of all cases of infertility in women (4–6). All these clinical manifestations impact the quality of life in EM patients, which creates a great physical and psychological burden. The mechanism underlying infertility in women with EM has not been established. Hormonal disorders, abnormal peritoneal function, distorted pelvic anatomy, and multiple repeat cesarean deliveries have been proposed to be involved in the pathogenesis of EM and infertility (7). In recent years, with the increasing number of multiple repeat cesarean deliveries, the incidence of EM is increasing year-after-year. Currently, pharmacologic treatment and surgery are the primary strategies for management of EM. Surgery is considered the most effective way to cure EM, especially for women with moderate and severe EM [revised American Fertility Society (rAFS) score III and IV, respectively] (8, 9).

Surgical treatment of EM includes traditional laparotomy (LT) and laparoscopic surgery (LS). The role of LS in improving pregnancy rates in women with EM is controversial, especially when compared to LT (10, 11). In the current study, LS is compared with LT to determine the LS clinical effect and improvement of infertility in patients with EM.

## Materials and methods

### Patients

Two hundred twenty-six patients with EM who were treated at the Third Hospital of Shijiazhuang City from June 2011 to June 2013 were enrolled in this retrospective study. This study was approved by the Ethics Committee of the Third Hospital of Shijiazhuang City (approval number: 2018-025). Signed informed consent was obtained from all participants. The characteristics of the patients are shown in Table 1.

**Table 1:** Demographic features of all subjects.

	Laparoscopic surgery			Laparotomy (*n* = 50)	*P* ^a^
	Cystectomy (*n* = 58)	Bipolar electrocoagulation forceps (*n* = 64)	Adnexectomy (*n* = 54)		
Age (years)	34.26 ± 6.56	32.95 ± 7.40	33.07 ± 6.11	33.94 ± 5.93	0.62
BMI (kg/m^2^)	22.85 ± 1.47	22.40 ± 1.44	22.67 ± 1.46	22.44 ± 1.26	0.41
Previous pregnancies (%)					>0.05
0	23 (39.66%)	28 (43.75%)	26 (48.15%)	29 (58.00%)	0.08
≥1	35 (60.34%)	36 (56.25%)	28 (51.85%)	21 (42.00%)	0.08
Infertility duration (years)	3.27 ± 0.93	3.29 ± 0.93	3.16 ± 0.88	3.58 ± 0.87	
rAFS stage					>0.05
I	22 (37.93%)	27 (42.19%)	23 (42.59%)	19 (38.00%)	0.75
II	13 (22.41%)	14 (21.88%)	17 (31.48%)	14 (28.00%)	0.72
III	16 (27.59%)	13 (22.41%)	12 (22.22%)	12 (24.00%)	1.0
IV	7 (12.07%)	10 (15.63%)	2 (3.70%)	5 (10.00%)	1.0
CA-125 (µg/mL)	45.06 ± 25.54	43.60 ± 22.47	33.23 ± 22.18	33.96 ± 22.08	0.67

BMI, body mass index; rAFS, revised American Fertility Society.

^a^ P: laparoscopic surgery group vs. laparotomy group.

### Inclusion and exclusion criteria

The inclusion criteria were as follows: (1) between 20 and 45 years of age; (2) laparoscopic-diagnosed EM; (3) moderate-to-severe endometrial-related pain, irregular menstruation, painful sexual intercourse, or dysmenorrhea, with a tendency to worsen progressively; (4) surgical treatment of EM included traditional LT and LS.

The exclusion criteria were as follows: (1) other chronic pain syndrome which requires chronic analgesics or chronic therapy; (2) current history of undiagnosed abnormal genital bleeding.

### Surgical procedure

All patients were intubated and general anesthesia was administered. When anesthesia was established, the surgical procedure was performed.

The LS group (*n* = 176) included three subgroups as follows: (1) The ovarian cystectomy group (*n* = 58), in which the varian cysts were peeled away and one side was treated first, followed by the other side (12). Of the 58 patients, 48, including 20 infertility patients, had desired fertility. (2) Bipolar electrocoagulation forceps (*n* = 64) were not used for patients in whom the Dow cavity was not present or dense adhesions existed (13). The entire Dow chamber was first isolated, followed by bipolar electrocoagulation of ectopic lesions. There were 54 cases, including 24 infertility patients, with desired fertility. (3) The adnexectomy group (*n* = 54) consisted of patients with multiple cysts on one side of the uterus who underwent adnexectomies (14). Forty-six of the patients, including 18 infertility patients, had desired fertility. A total of 148 patients with desired fertility, including 62 infertility patients, were treated by LS.

The LT group (*n* = 50) underwent pelvic adhesiolysis with fulguration of the visually discernible lesions scattered within the pelvis (<0.5 cm) using conventional methods (15). There were 40 patients in this group (including 20 with infertility) who had desired fertility.

### Outcomes measured

Postoperative clinical outcomes, including dysmenorrhea, dyschezia, dyspareunia, pelvic pain, abnormal uterine bleeding, and dysuria, were recorded. The operative duration, average length of stay in the hospital, and intraoperative blood loss were noted. Patients were followed for 1 year to investigate postoperative pregnancy rates.

### Statistical analysis

The demographic features, postoperative symptoms, and reproductive outcomes were analyzed using SPSS 17.0 software. Measurement data [age, body mass index (BMI), duration of infertility, CA-125 level, operative time, average length of hospital stay, and blood loss] are expressed as the mean ± standard deviation (SD). A *t*-test was used to compare the mean difference between the LS and LT groups. Multivariables were analyzed with one-way ANOVA followed by the Bonferroni test. Count data, such as previous pregnancies, rAFS stage, dysmenorrhea, dyschezia, dyspareunia, pelvic pain, abnormal uterine bleeding, dysuria, and percentage pregnant, are expressed as n (%), and the differences between the two groups were analyzed using the *χ*^2^ Pearson test. *P* < 0.05 was considered statistically significant.

## Results

### Baseline characteristics of included patients

Table 1 shows the demographic and baseline reproductive characteristics of all patients. The ages of the participants ranged from 22 to 45 years, with a mean age of 33.6 years. One hundred six patients (46.9%) were nulliparous, while the remaining 53.1% had ≥1 children. There were 91, 58, 53, and 24 patients with rAFS stage I, II, III, and IV endometriosis, respectively. The patients in the LS and LT groups had infertility for a mean of 3.38 ± 0.92 and 3.58 ± 0.87 years, respectively.

Unilateral ovarian enlargement was noted in 70 patients, bilateral ovarian enlargement in 36 patients, and depression in 26 patients. There were no differences among patients in the LS and LT groups with respect to age, BMI, previous pregnancies, infertility duration, rAFS stage, and the level of CA-125 (*P* > 0.05). Among 226 patients, 188 had desired fertility (106 infertility patients, 82 of whom had desired fertility).

### Surgical procedures and postoperative symptoms

**Figure 1:**
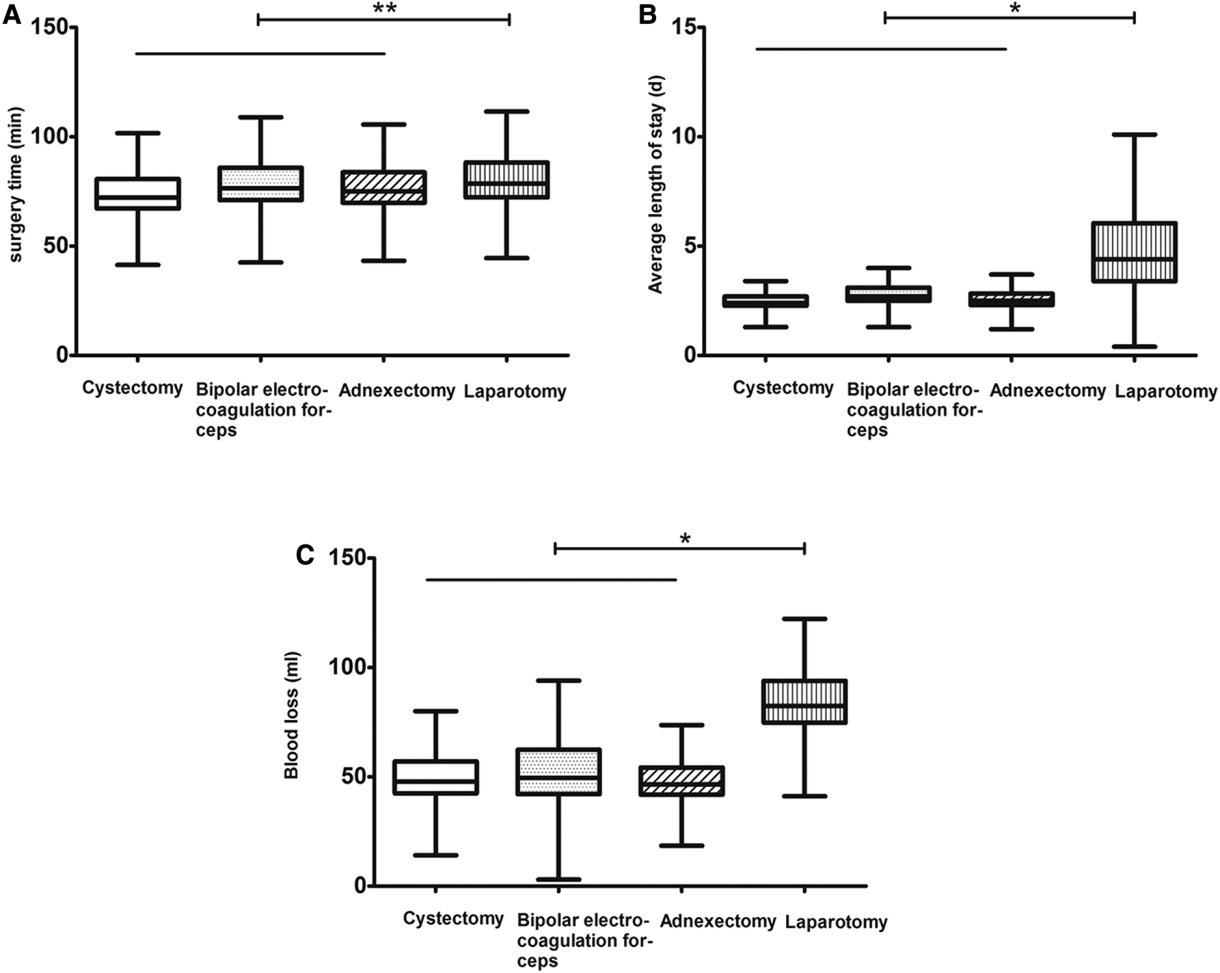
Features of operative time (**A**), average length of hospital stay (**B**), and blood loss (**C**) during the procedures. Cystectomy is indicated by a clear symbol, bipolar electrocoagulation forceps by a punctate symbol, adnexectomy by a slanted symbol, and laparotomy by a vertical symbol. *indicates *P* < 0.05 and **indicates *P* > 0.05.

The surgical procedures and postoperative symptoms were recorded (Table 2). Figure 1 depicts the parameters of operative time, average length of hospital stay, and blood loss. After treatment, all lesions, regardless of size, were essentially eliminated, and the resected lesions were confirmed to be EM based on pathologic evaluation. The operative times for the LS and LT groups were not significantly different (75 ± 11 vs. 79 ± 12 min, *P* > 0.05; Figure 1A); however, there were statistically significant differences in the average length of hospital stay (2.5 ± 0.5 vs. 4.6 ± 2.0 days, *P* = 0.00; Figure 1B) and blood loss (48.5 ± 13.4 vs. 83.2 ± 13.4 ml, *P* = 0.00; Figure 1C) between the two groups. The incidence of postoperative dysmenorrhea (10.23% vs. 24%, *P* = 0.017), dyschezia (13.64% vs. 30%, *P* = 0.011), dyspareunia (3.98% vs. 14%, *P* = 0.017), pelvic pain (16.48% vs. 30%, *P* = 0.043), and dysuria (11.93% vs. 26%, *P* = 0.023) was lower in the LS group than the LT group (Table 2).

**Table 2:** Surgical procedures and postoperative symptoms for laparoscopic surgery and laparotomy for endometriosis.

	Laparoscopic surgery			Laparotomy (*n* = 50)	*P* ^a^
	Cystectomy (*n* = 58)	Bipolar electrocoagulation forceps (*n* = 64)	Adnexectomy (*n* = 54)		
Surgical procedures					
Operative time (min)	72 ± 11	76 ± 12	75 ± 11	79 ± 12	0.08
Average length of hospital stay (day)	2.42 ± 0.39	2.71 ± 0.51	2.53 ± 0.44	4.63 ± 1.95	0.00
Blood loss (ml)	50.5 ± 22.6	53.2 ± 27.4	48.7 ± 15.6	83.2 ± 13.4	0.00
Postoperative symptoms					
Dysmenorrhea	6 (10.34%)	6 (9.38%)	6 (11.11%)	12 (24.00%)	0.017
Dyschezia	7 (12.07%)	8 (12.5%)	9 (16.67%)	15 (30.00%)	0.011
Dyspareunia	3 (5.17%)	2 (3.13%)	2 (3.70%)	7 (14.00%)	0.017
Pelvic pain	10 (17.24%)	8 (12.50%)	11 (20.37%)	15 (30.00%)	0.043
Abnormal uterine bleeding	4 (6.89%)	1 (1.54%)	2 (3.70%)	4 (8.00%)	0.266
Dysuria	9 (15.52%)	5 (7.69%)	7 (12.96%)	13 (26.00%)	0.023

^a^ P: laparoscopic surgery group vs. laparotomy group.

### Reproductive outcomes

**Figure 2:**
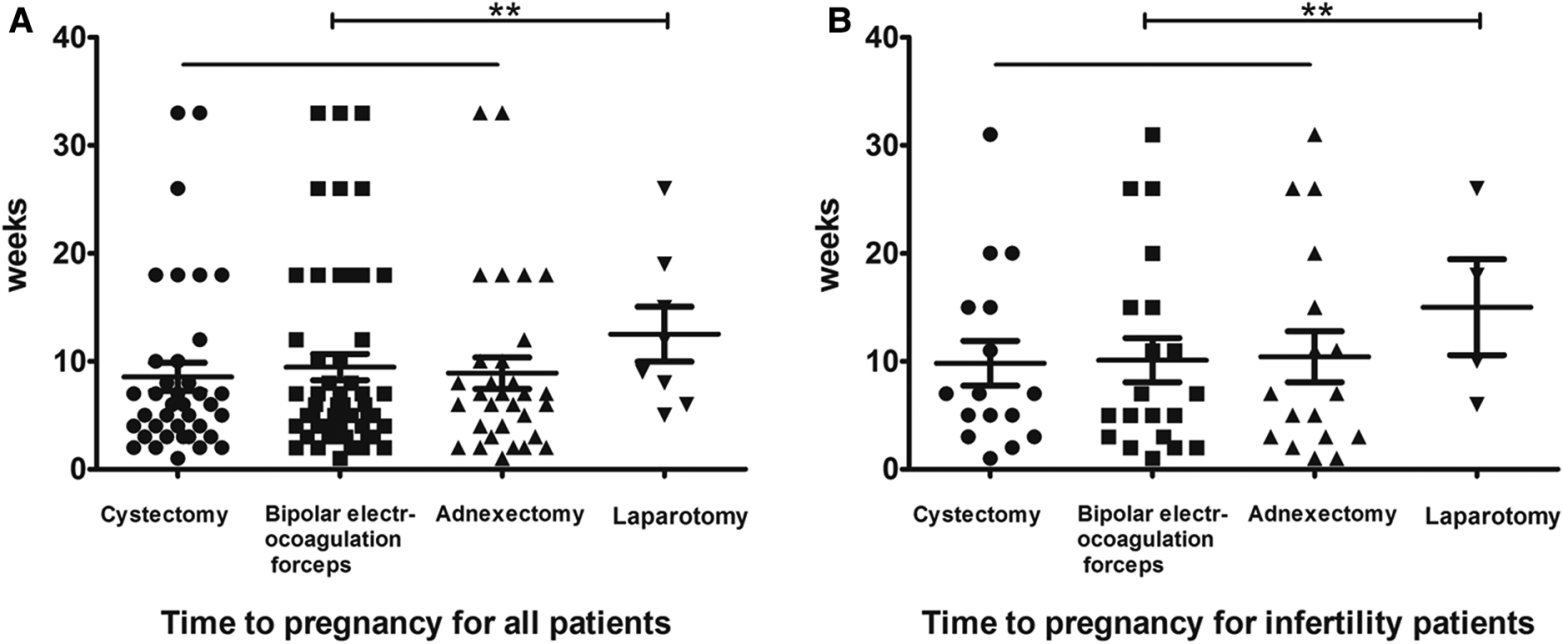
Time to pregnancy for all patients (**A**) and infertility patients (**B**). Cystectomy is indicated by bold dots, bipolar electrocoagulation forceps by square dots, adnexectomy by erected triangle dots, and laparotomy by inverted triangle dots. **indicates *P* > 0.05.

**Figure 3:**
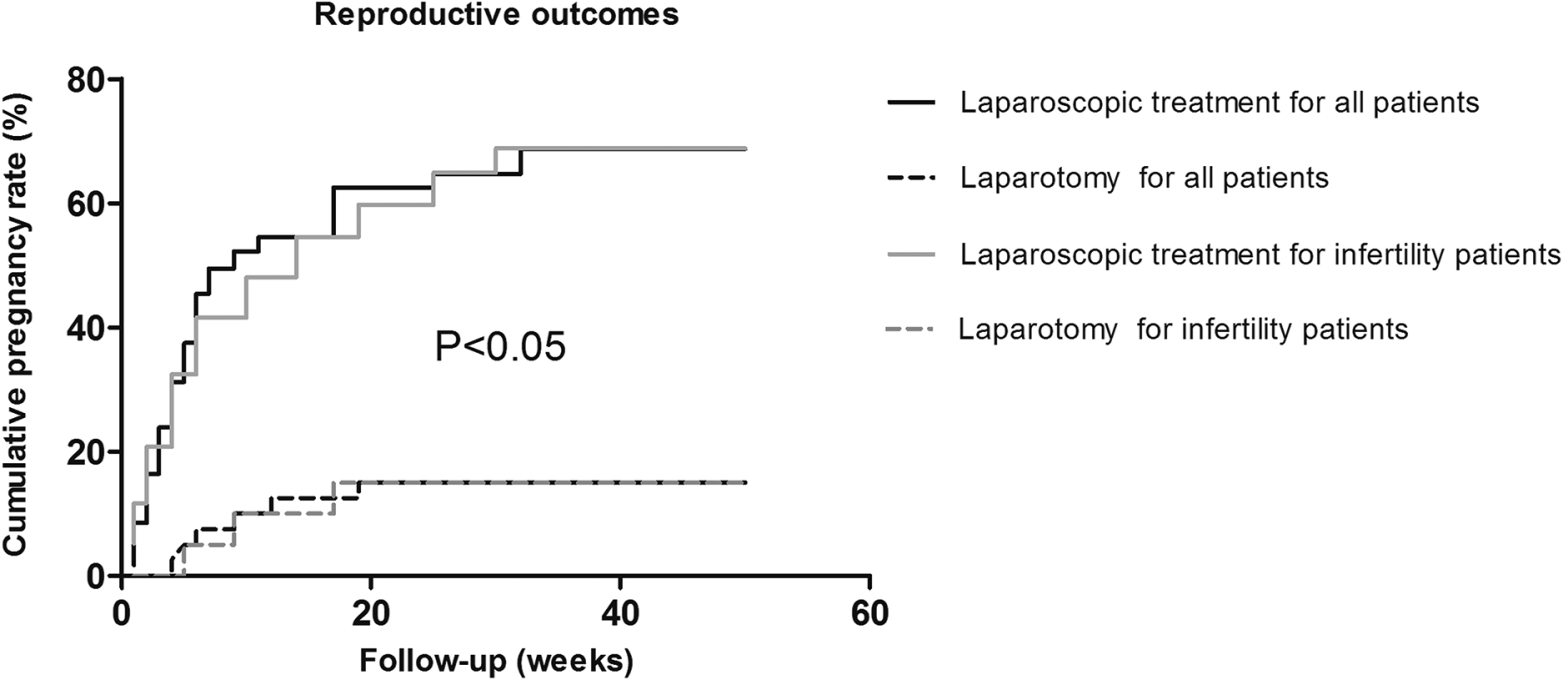
The cumulative pregnancy rates in the laparoscopic group and the traditional laparotomy group. Laparoscopic treatment is indicated by a solid black line for all patients and a solid gray line for infertility patients, and laparotomy is indicated by a dashed black line for all patients and a dashed gray line for infertility patients.

One hundred eighty-eight patients with desired fertility were followed 1 year postoperatively to confirm clinical pregnancy rates. The fertility results are shown in Table 3. Of the 148 patients who underwent LS, 101 achieved pregnancies (pregnancy rate = 68.24%). Of the 40 patients who underwent LT, 6 achieved pregnancies (pregnancy rate = 15%). The average time to pregnancy was 9.0 weeks in the LS group compared to 12.5 weeks in the LT group; there were statistically significant differences between the two groups (Figure 2A). Similar results were noted in infertility patients. Of the 82 infertility patients with desired fertility, 62 were treated by LS; 43 achieved pregnancies 1 year after surgery for a pregnancy rate of 69.35%. Of the 20 patients who underwent LT, only 3 achieved pregnancies, for a pregnancy rate of 15.00%. The mean time to pregnancy was 10.1 weeks in the LS group compared to 15.0 weeks in the LT group (Figure 2B). There was a statistically significant difference in the cumulative pregnancy rate between the LS and traditional LT groups (Figure 3).

**Table 3:** Reproductive outcomes at 1-year follow-up evaluation.

		Laparoscopic surgery			Laparotomy	*P* ^a^
		Cystectomy	Bipolar electrocoagulation forceps	Adnexectomy		
Patients with desired fertility (*n* = 188)	Percent pregnant (%)	31 (64.58%)	43 (79.63%)	27 (58.69%)	6 (15.00%)	
	Total (%)	101 (68.24%)			6 (15.00%)	0.00
	Average time to pregnancy (weeks)	8.6	9.5	8.9	12.5	
	Total (weeks)	9.0			12.5	0.251
Infertile patients with desired fertility (*n* = 82)	Percent pregnant (%)	14 (70.00%)	17 (70.83%)	12 (66.67%)	3 (15.00%)	
	Total	43 (69.35%)			3 (15.00%)	0.00
	Average time to pregnancy (weeks)	9.8	10.1	10.4	15	
	Total (weeks)	10.1			15	0.296

^a^ P: laparoscopic surgery group vs. laparotomy group.

## Discussion

In this study, LS for EM achieved better clinical outcomes than LT. There was no statistical difference in operative times between the LS and LT groups with respect to cystectomies, bipolar electrocoagulation forceps fulguration, and adnexectomies. The length of hospital stay and blood loss were significantly different between the LS and LT groups; however, none of the patients had adverse reactions.

The incidence of postoperative symptoms, such as dysmenorrhea, dyspareunia, pelvic pain, and dysuria, in the LS group was also significantly lower than the LT group. In contrast, the incidence of most postoperative symptoms in the LS group was lower than the LT group. Normal physiologic functions were restored within 24 h after LS and patients returned to work within 1–2 weeks. LS has obvious advantages with respect to surgical trauma, postoperative recovery, and safety, which allows patients to have smaller wounds, less blood loss, and shorter hospital stays.

The conception rate of patients with desired fertility in the LS (*n* = 62) and LT groups (*n* = 20) were also compared. The postoperative pregnancy rate in the LS group, including the postoperative pregnancy rate of infertility patients, was significantly higher than the LT group, indicating that LS treatment of EM can significantly improve infertility. Schippert et al. (16) reported that 74.3% (52/70) of women became pregnant after LS for EM, and 61.3% (19/31) became pregnant after LT; 42.1% (8/19) became pregnant after switching from LS to LT. There was a statistically significant correlation between the postoperative conception rate and surgical approach (*P* = 0.00893).

In recent years, with a clear upward trend in the incidence of EM, there has been growing concern among women of childbearing age (17, 18). Because EM is not sensitive to medications, surgery is the primary treatment modality. LS is considered the gold standard for determining the extent of lesions in patients at different times because of its advantages in assisting physicians in diagnosis and also as a means to treat the disease. LS also has a magnification feature that can clearly show the distribution of small lesions in the pelvis, detect early cases of EM, achieve accurate and thorough surgery, reduce misdiagnosis and residual lesions, relieve pain, and improve surgical outcomes. Compared with LT, LS fully demonstrates its advantages with respect to surgical trauma, postoperative recovery, and safety. Because LS is used clinically, LS has been approved by the majority of clinicians and patients (19).

This study showed that the treatment of EM with LS was associated with fewer adverse effects, faster patient recovery, and better postoperative pregnancy outcomes. To ensure good treatment efficacy and reduce surgical accidents, medical staff should take detailed medical histories from patients, perform all preoperative examinations, clarify the surgical indications and contraindications, and make comprehensive assessments of potential intraoperative problems.

This study had several limitations. The LS approach is usually regarded as safe, but as with any surgical treatment, there are risks, such as internal bleeding, herniation at the incision, infection, and injury to blood vessels or other organs, including the stomach, intestines, and bladder. Furthermore, the results of pain and fertility following LS should be investigated further according to the rAFS stage. Further studies should be based on multicenter randomized controlled trials and large samples.

## Conclusions

Compared with LT, LS significantly reduced pain and improved the quality of life in women with EM. These results can provide a reference for the clinical diagnosis and treatment of EM.

## Data Availability

The original contributions presented in the study are included in the article/Supplementary Material, further inquiries can be directed to the corresponding author.
